# Exploring the spiritual foundations of public health leadership

**DOI:** 10.3389/fpubh.2023.1210160

**Published:** 2023-10-25

**Authors:** Howard K. Koh, Cathy C. Tso, Cyra Perry Dougherty, Emily E. Lazowy, Chelsea P. Heberlein, Fawn A. Phelps

**Affiliations:** ^1^Harvard T.H. Chan School of Public Health, Boston, MA, United States; ^2^Harvard Kennedy School, Cambridge, MA, United States

**Keywords:** spirituality, leadership, public health, education, meaning, purpose, resilience

## Abstract

The Covid-19 pandemic has laid bare the challenges of public health leadership. Faced with criticism, threats, and even violence, many public health leaders have left the field. A healthier future for the nation may well rest on training aspiring public health leaders to build deeper capacity for perseverance, healing, and resilience. Reflecting the growing experience of a team of public health educators at the Harvard T.H. Chan School of Public Health (Harvard Chan), this article offers recommendations for public health schools to recognize, and incorporate into leadership education, themes of spirituality—ie, the way people seek ultimate meaning and purpose and deep connectedness to something larger than themselves. Doing so can serve as a foundation for the lifelong journey of leadership. Over the past decade, Harvard Chan has incorporated meaning, purpose, and connectedness themes to complement more traditional coursework addressing research and translation. While many established leadership frameworks address the “what” and “how” of career development, the spirituality framework can support aspiring leaders to more fully understand their “why” and its alignment with challenging work. Such a deeply personal topic, traditionally kept private, has been shared and nurtured in Harvard Chan classrooms through a range of pedagogical strategies including personal reflection, one-on- one coaching, experiential learning, case discussions, and candid conversations with public health leaders. By encouraging a values-based foundation for decision-making in crises and difficult leadership moments, such grounding can help aspiring leaders navigate the challenges of public health leadership that inevitably lie ahead.

## Introduction

Covid-19 has laid bare the challenges of public health leadership. Through the pandemic, leaders struggling to provide health guidance to an anxious public have endured searing experiences involving harassment, threats, and even violence (1). Such unsettling and even traumatic episodes have contributed to a steady exodus from the field (2, 3). According to some estimates, at least 500 top national and local public health leaders had, just a year into the pandemic, resigned or been fired (4). Moreover, almost half of the state and local public health workers left their jobs (4).

The nation’s ability to not only weather such health crises but also build better systems for the future may well rest on explicit training to build motivation, stamina, and resilience for aspiring leaders (5). In this article, we describe how one public health school has attempted to achieve such goals by steadily incorporating themes of meaning, purpose, and connectedness into leadership education. Such themes can be broadly captured under the term “spirituality,” defined in an international consensus conference as “a dynamic and intrinsic aspect of humanity through which persons seek ultimate meaning, purpose, and transcendence, and experience relationship to self, family, others, community, society, nature, and the significant or sacred” (6). For some, spirituality involves connection to organized faith traditions. For others, it involves connection with a cause or some significant entity that is greater than themselves. People can be spiritual but not religious; indeed, those who identify as atheists and agnostics can be, according to this definition, highly spiritual. But for all people, such themes are deeply personal, often rooted in cultural upbringing, and/or associated with major life experiences that shape values and beliefs (7).

To date, public health leadership educators have largely overlooked these themes. A review of the literature shows virtually no attention to the spirituality theme as part of public health leadership education. Over the past decade, however, our teaching team at the Harvard T.H. Chan School of Public Health (Harvard Chan) has steadily curated a curriculum that explicitly calls out meaning, purpose, and connectedness as fundamental to leadership. In this article we briefly describe this approach and its rationale, together with reflections on ways such spiritual dimensions might complement other leadership competencies more frequently noted in the published literature (8).

## Public health leadership education and spirituality

Leadership poses extraordinary challenges for public health, a field in which highly complex and seemingly unsolvable problems characteristically abound. Often enormous in scale and impacting populations broadly, public health challenges usually, if not inevitably, fall outside the control of a single authority in volatile and politicized setting that involve passionate stakeholders, powerful vested interests (9) and controversies about how best to distribute finite resources to address infinite needs (10). Moreover, the field, by definition, requires performing “on stage,” with leaders subjected to intense scrutiny and where everything is “fair game for comment, criticism, and interpretation (or misinterpretation)” (11).

Despite these challenges, only 55% of accredited public health schools offer any kind of course on public health leadership (12). Even when they do, the emphasis is more often, as noted by Ganz, on external strategy (“head”) and action (“hands”), and less often on the personal narrative of motivation (“heart”) (13). However, Sinek advocates “starting with why”— ie, “working from the inside out”—as a way for aspiring leaders to ground their work and actions in a foundation of meaning, purpose, and connectedness (14). Guiding one’s work in this way also equips students to better connect with the “who,” ie, the “Ps” of public health— that include policymakers, purchasers, and the press, not to mention passionate advocates and penurious budget officials (5, 9, 15). In this way, leaders can better recognize the source of both their motivations and biases, persevere through crises (9), include other voices in collective action, navigate uncertainty, and ultimately find renewed and deeper meaning and purpose. Solidifying this foundation readies them, in a way current training systems may not, to lead teams, organizations, and systems in whatever the future befalls them.

A recent national expert panel has similarly urged more integration of the worlds of spirituality and health, given growing evidence of their strong links (16). One relevant meta- analysis of 10 prospective studies of ~136,000 participants associated a high sense of purpose in life with a reduced risk for all-cause mortality and cardiovascular events (adjusted pooled relative risk = 0.83) (17). Another recent comprehensive study by Balboni et al. (16) analyzed the century’s nearly 600 highest-quality related studies (2000–2022) on the subject and associated spirituality with greater years of life and health-related quality of life.

Public health can also learn from other sectors that have demonstrated the value of incorporating spirituality themes in enhancing leadership. In business, for example, Fry and colleagues have found their model of spiritual leadership in the workplace linked to greater organizational commitment and productivity (18) through a heightened sense of “engagement of the whole person,” belonging, and life satisfaction (19). In medical education, the concept of finding a sense of professional belonging has been called “socialization into profession” (20). The field of public health may be particularly amenable to such themes, as it inherently features a sense of mission and calling, especially through difficult times like a pandemic.

Encouraging public health students to share the origins of their “why” in classroom discussion may free them to let down their guard, “bring their whole self” to discussions, and convey authenticity and transparency. It can also provide a means to connect to a shared sense of purpose, promote empathy, build genuine curiosity and appreciation for others’ motivations, and cultivate the sense of interdependence so elusive in the vitriol of the pandemic (21). This approach has the potential to advance for students the overarching educational goal of fostering “confidence in their authentic self rather than trying to adhere to a prescribed definition of leadership” (22).

Addressing the “why” in the classroom to understand diverse views and belief may lead some students to share strong personal beliefs of faith and religion. For some listeners, that may trigger discomfort and concerns about inclusivity and vulnerability; indeed, explicit expressions of religion may be helpful for many while hurtful for many others. However, it can be argued that such sharing in a student-centered approach can offer healing in the midst of difficult conversations regarding issues of pain and even trauma. Frankl has noted, that “life is never made unbearable by circumstances, but only by lack of meaning and purpose” (23) and as noted by Miller, Gupta, and others, many religions highlight themes common in public health, regardless of specific faith (24, 25). From his personal teaching experience, the lead author of this paper regularly asks students to reflect on if, and how, spirituality has impacted their public health leadership journey. In general, students appreciate direct conversations about spirituality, although some have also been explicit about how religion has negatively impacted their lives. Meanwhile, theologians from different denominations regularly emphasize themes of human interconnectedness, service to others, and discovering deeper meaning in difficult times. Thich Nhat Hanh noted “our own life has to be our message” (26). Martin Luther King noted that we are all a part of “…an inescapable network of mutuality, tied in a single garment of destiny. Whatever affects one directly, affects all indirectly” (27) and William Sloane Coffin urged that we should “care most for those society counted least and put last” (28).

## Integrating spirituality themes into public health leadership education at Harvard Chan

Over the past decade, Harvard Chan has encouraged students to clarify their meaning, purpose, and sense of connectedness as a foundation for their professional journey.

Introduction to the topic starts on the opening day of orientation, where faculty note that a sense of vocation and mission can be integral to the profession. In fact, in an orientation poll, when students are asked to choose between “I never thought I would be attending a public health school but here I am” versus “I always knew I wanted to attend a public health school,” the results, routinely 2:1 in favor of the former, regularly spark discussions about calling and vocation as part of a public health career. Students can dig deeper in the related orientation workshop “Calling to Public Health,” where they share values and beliefs that fuel their professional aspirations.

Harvard Chan also offers additional optional opportunities for students to explore their leadership “why.” The non-credit Public Health Leadership Lab, a semester-long co- curricular program with weekly workshops, provides students a venue to develop a greater understanding of their motivations through shared reflection. Such workshops focus on attentive listening to self and others as a basis for understanding organizations and systems. Faculty model how listening can serve as a powerful method of staying connected to one’s purpose and that of others. Interested students can also seek peer coaching as well as executive coaching by seasoned mentors. Moreover, several elective leadership courses allow students to pose questions about motivation, meaning and purpose to guest speakers with storied public health careers captured in case studies. Qualitative program evaluation through classroom feedback surveys and course evaluations are used for continuous curriculum revision.

In addition, the Doctor of Public Health (DrPH) program starts each cohort with a year-long development course on “personal mastery,” one of five disciplines identified by organizational leadership expert Peter Senge as essential to leading large-scale systems change (29). Personal mastery emphasizes clarifying one’s vision, values, strengths, and weaknesses through the lens of connection to purpose, self, and others. Other DrPH courses focus on leading teams and addressing systems change. And the culminating doctoral project requires a leadership development statement grounded in students’ purpose and values.

In such educational efforts, students can then integrate inquiry and reflection about meaning, purpose, and connectedness into learning from established leadership theories. Pedagogical methods, which include regular contemplative practices that encourage mindfulness (awareness and attention), journaling, listening, and structured dialog are drawn from a diverse and expansive collection of contemplative practices from wisdom traditions, adult development research, leadership development pedagogy, and social movement organizing practices; works included are by experts such as Barry (30), Brown (31), Freire (32), Hemphill (33), Kabat-Zinn (34), Kegan and Lahey (35, 36), Kolb (37), Palmer (38), and Scharmer (39). Many established leadership development frameworks lend themselves to such integration, such as Goleman’s Emotional Intelligence (40), George’s Authentic Leadership Framework (41), Heifetz’s and Linsky’s Adaptive Leadership Framework (42), Edmondson’s work on teaming and psychological safety (43), and Bennis’ “Crucibles of Leadership” (44).

Bennis, for example, emphasizes that the best leaders are ones that can reframe crucible experiences—i.e., intense, unplanned and traumatic experiences that make one deeply question one’s values and identity—to emerge stronger than before. Such themes reinforce writings by Nouwen who celebrated that the search for deeper meaning during tough life experiences can motivate wounded people to serve as “wounded healers” (11, 45). Such reframing additionally allows leaders to stay grounded in mission while developing the “existential flexibility” (46) essential for the challenges ahead. Such skills can complement the updated 2022 Lancet Commission Report on the future of public health education, which underscores the need for leaders who can leverage a range of competencies as part of their “education for life” (8).

## Discussion and recommendations

*“I used to think that leadership just required learning about leadership skills, but now I think that leadership development is a lifelong process that requires active and ongoing reflection.”* (47)

*-Harvard T.H. Chan Student Feedback, May 2022 Program Evaluation.*

Harvard Chan’s leadership curriculum, while evolving over a decade, is still embryonic. To our knowledge, few, if any, published articles explore the link between public health leadership and spirituality. We hope that sharing our perspectives will prompt national dialog on how to further explore and support such fundamental and deeply personal themes for future public health leaders.

To promote future dialog, we propose that schools of public health consider the following recommendations to prepare leaders:

- Provide more opportunities for self- and systems- awareness practices in public health leadership education.
- Build a classroom culture of belonging where students can comfortably share their inner selves and listen respectfully to others share theirs as a way of building empathy, respecting differences, and even managing difficult conversations.
- Offer experiential learning opportunities in which exploration of self, purpose, and relationships are prioritized as much as traditional instruction in research methods and theories.
- Invite public health leaders to speak openly about their inner lives, their sources of support in navigating uncertainty and complexity, and their practices for healing.
- Build more explicit competencies in clarifying and refining values, purpose, and motivations for public health leadership work. Adding attention to spirituality could be considered for incorporation into future CEPH public health accreditation related to leadership competencies (48).

## Conclusion

Students should not be left on their own to develop leadership skills essential to address pandemics and other highly complex challenges. Our experiences offer a way forward for public health schools to support students in exploring spirituality themes of ultimate meaning, purpose, and connectedness to something greater than themselves. In our view, such training has great potential to strengthen a foundation that encourages students to take the leap of faith that public health leadership necessarily entails.

## Data availability statement

The original contributions presented in the study are included in the article/supplementary material. Further inquiries can be directed to the corresponding author.

## Author contributions

HK led the coordination and writing of the piece and contributed subject matter expertise on all topics discussed in the paper and recommendations. CT, FP, and CD contributed extensively to the sections on spirituality and public health education, the curriculum at the Chan School and recommendations. EL and CH contributed to the sections about spirituality, leadership, and recommendations. They also led all fact-checking and references efforts. All authors contributed to the article and approved the submitted version.

## References

[1] Ward JA Stone EM Mui P Resnick B. Pandemic-related workplace violence and its impact on public health officials, March 2020–January 2021. Am J Public Health 1971. (2022) 112:736–46. doi: 10.2105/AJPH.2021.306649 PMC9010912 35298237

[2] Orr JM Leider JP Kuehnert P Bekemeier B. ANALYSIS: COVID-19 revealed shortcomings of the US public health system and the need to strengthen funding and accountability. Health Aff. (2023) 42:374–82. doi: 10.1377/hlthaff.2022.01234 36877906

[3] Leider JP Castrucci BC Robins M Hare Bork R Fraser MR Savoia E. The exodus of state and local public health employees: separations started before and continued throughout COVID-19: study examines state and local public health agency employees intent to leave or retire in 2017 with actual separations through 2021. Health Aff. (2023) 42:338–48. doi: 10.1377/hlthaff.2022.01251, PMID: 36877909

[4] Baker M Ivory D. Why public health faces a crisis across the U.S. New York Times (online). (2021).

[5] Koh HK. Educating future public health leaders. AJPH. 105:2385. doi: 10.2105/AJPH.2014.302385 PMC4339986 25706004

[6] Puchalski CM Blatt B Kogan M Butler A. Spirituality and health: the development of a field. Acad Med. (2014) 89:10–6. doi: 10.1097/ACM.0000000000000083 24280839

[7] Wigglesworth Cindy. Integral theory (also called AQAL theory) and its relationship to spiritual intelligence and the SQi assessment. Available at: https://deepchange.com/IntegralSpiritualIntelligence2011.pdf.

[8] Frenk J Chen LC Chandran L Groff EOH King R Meleis A. Challenges and opportunities for educating health professionals after the COVID-19 pandemic. Lancet. (2022) 400:1539–56. doi: 10.1016/S0140-6736(22)02092-X, PMID: 36522209 PMC9612849

[9] Koh HK Jacobson M. Fostering public health leadership. J Public Health. (2009) 31:199–201. doi: 10.1093/pubmed/fdp032 19451343

[10] Koh HK. Navigating government service as a physician. Health Educ Behav. (2016) 43:7–10. doi: 10.1177/1090198115614318 26747713

[11] Koh HK. The public health journey: the meaning and the moment. Health Educ Behav. (2013) 40:635–9. doi: 10.1177/1090198113501514 23988513

[12] Hertelendy AJ Chekijian S McNulty E Mitchell CL Grimes JO Durneva P. Crisis leadership: a case for inclusion in accredited master of public health program curricula. Public Health. (2022) 209:14–8. doi: 10.1016/j.puhe.2022.05.012 35749926

[13] Ganz M. Why David sometimes wins: leadership, organization, and strategy in the California farm worker movement. Oxford; New York: Oxford University Press; (2009). xvii–344.

[14] Sinek S. Start with why: how great leaders inspire everyone to take action. East Rutherford: Penguin Publishing Group (2009).

[15] Koh H. Leadership in public health. J Cancer Educ. (2009) 24:11–8. doi: 10.1080/08858190903400385 PMC7097219 20024817

[16] Balboni TA VanderWeele TJ Doan-Soares SD Long KNG Ferrell BR Fitchett G. Spirituality in serious illness and health. J Am Med Assoc. (2022) 328:184–97. doi: 10.1001/jama.2022.11086 35819420

[17] VanderWeele TJ Balboni TA Koh HK. Health and spirituality. J Am Med Assoc. (2017) 318:519–20. doi: 10.1001/jama.2017.8136 28750127

[18] Fry LW. Toward a theory of spiritual leadership. Leadersh Q. (2003) 14:693–727. doi: 10.1016/j.leaqua.2003.09.001

[19] Fry LW Latham JR Clinebell SK Krahnke K. Spiritual leadership as a model for performance excellence: a study of Baldrige award recipients. J Manag Spiritual Relig. (2017) 14:22–47. doi: 10.1080/14766086.2016.1202130

[20] Dopelt K Davidovitch N Yahav Z Urkin J Bachner YG. Reducing health disparities: the social role of medical schools. Med Teach. (2014) 36:511–7. doi: 10.3109/0142159X.2014.891006 24796237

[21] Koh HK Nowinski JM. Health equity and public health leadership. Am J Public Health. (2010) 100:S9–S11. doi: 10.2105/AJPH.2010.191379 20147661 PMC2837440

[22] Lachance JA Oxendine JS. Redefining leadership education in graduate public health programs: prioritization, focus, and guiding principles. Am J Public Health. (2015) 105:S60–4. doi: 10.2105/AJPH.2014.302463 25706021 PMC4339997

[23] Frankl VE. Man’s search for meaning. Rev. and updated ed. New York: Washington Square Press/Pocket Books (1985). 221 p.

[24] Gupta A. Bridges across humanity. Cambridge, MA: Rupa Publications (2023).

[25] Miller L. The awakened brain: the new science of spirituality and our quest for an inspired life. 1st ed. New York: Random House (2021).

[26] Thich Nhat Hanh. The world we have: a Buddhist approach to peace and ecology. Cambridge, MA: Parallax Press (2008).

[27] King ML. Letter from a Birmingham jail. (1963). Available at: https://www.africa.upenn.edu/Articles_Gen/Letter_Birmingham.html (Accessed March 16, 2023).

[28] Coffin WS Jr. Letters to a young doubter. 1st ed. Westminster: John Knox Press (2005).

[29] Senge PM. The fifth discipline: the art and practice of the learning organization. New York: Doubleday/Currency (1990). 444 p.

[30] Barry L. Syllabus. Montreal, Canada: Drawn & Quarterly (2019).

[31] Brown AM. Emergent strategy: shaping change, changing worlds. Edinburgh, United Kingdom: AK Press (2017).

[32] Freire P. Pedagogy of the oppressed. 30th ed. New York: Continuum (2000).

[33] Hemphill P. *The wisdom of process*. In: T. Burke, B. Brown editors. You are your best thing: vulnerability, shame resilience, and the black experience: an anthology. 1st edn. New York: Random House (2021). p. 43–53.

[34] Kabat-Zinn J. Mindfulness for beginners: reclaiming the present moment--and your life. Boulder, CO: Sounds True (2016).

[35] Kegan R. In over our heads: the mental demands of modern life. Cambridge, MA: Harvard University Press (1994).

[36] Kegan R. An everyone culture: becoming a deliberately developmental organization. First eBook ed. Boston, Massachusetts: Harvard Business Review Press (2016).

[37] Kolb DA. Experiential learning: experience as the source of learning and development. Englewood Cliffs, NJ: Prentice-Hall (1984). xiii+256 p.

[38] Palmer PJ. A hidden wholeness: the journey toward an undivided life: welcoming the soul and weaving community in a wounded world (1st Edn.). San Francisco, CA: Jossey- Bass; (2004). xiii–208.

[39] Scharmer O. The essentials of theory U: core principles and applications. Oakland: Berrett-Koehler Publishers (2018).

[40] Goleman D. Focus: the hidden driver of excellence. 1st ed. New York, NY: Harper (2013).

[41] George B. Authentic leadership: rediscovering the secrets to creating lasting value (1st Edn.). San Francisco: Jossey-Bass; (2003). xix–217.

[42] Heifetz RA Linsky M Grashow A. The practice of adaptive leadership: tools and tactics for changing your organization and the world. Boston, United States: Harvard Business Review Press (2009).

[43] Edmondson A. Psychological safety and learning behavior in work teams. Adm Sci Q. (1999) 44:350–83. doi: 10.2307/2666999

[44] Bennis WG. The seven ages of the leader. IEEE Eng Manag Rev. (2004) 32:45–5. doi: 10.1109/EMR.2004.25049 14723176

[45] Nouwen HJM. Ministry by a lonely minister: the wounded healer. In: The wounded healer. 1st edn. New York: DoubleDay (1972). p. 79–96.

[46] Sinek S. The infinite game. London, England: Portfolio Penguine (2020).

[47] Tso C. [Unpublished raw data from Public Health Leadership Lab Year-End Evaluation Survey]. Harvard T.H. Chan School of Public Health. (2022).

[48] Council on Education for Public Health (CEPH). Accreditation criteria: schools of public health & public health programs. Silver Spring, MD. (2021). p. 18. (Accessed September 29, 2023).

